# DeephageTP: a convolutional neural network framework for identifying phage-specific proteins from metagenomic sequencing data

**DOI:** 10.7717/peerj.13404

**Published:** 2022-06-08

**Authors:** Yunmeng Chu, Shun Guo, Dachao Cui, Xiongfei Fu, Yingfei Ma

**Affiliations:** 1 Shenzhen Key Laboratory of Synthetic Genomics, Guangdong Provincial Key Laboratory of Synthetic Genomics, CAS Key Laboratory of Quantitative Engineering Biology, Shenzhen Institute of Synthetic Biology, Shenzhen Institutes of Advanced Technology, Chinese, Shenzhen, Guangdong, P.R. China; 2 Department of Bioengineering and Biotechnology, Huaqiao University, Xiamen, Fujian, P.R. China

**Keywords:** Convolutional neural network, Deep learning, Phage-specific protein, Phage, Metagenomics

## Abstract

Bacteriophages (phages) are the most abundant and diverse biological entity on Earth. Due to the lack of universal gene markers and database representatives, there about 50–90% of genes of phages are unable to assign functions. This makes it a challenge to identify phage genomes and annotate functions of phage genes efficiently by homology search on a large scale, especially for newly phages. Portal (portal protein), TerL (large terminase subunit protein), and TerS (small terminase subunit protein) are three specific proteins of Caudovirales phage. Here, we developed a CNN (convolutional neural network)-based framework, DeephageTP, to identify the three specific proteins from metagenomic data. The framework takes one-hot encoding data of original protein sequences as the input and automatically extracts predictive features in the process of modeling. To overcome the false positive problem, a cutoff-loss-value strategy is introduced based on the distributions of the loss values of protein sequences within the same category. The proposed model with a set of cutoff-loss-values demonstrates high performance in terms of Precision in identifying TerL and Portal sequences (94% and 90%, respectively) from the mimic metagenomic dataset. Finally, we tested the efficacy of the framework using three real metagenomic datasets, and the results shown that compared to the conventional alignment-based methods, our proposed framework had a particular advantage in identifying the novel phage-specific protein sequences of portal and TerL with remote homology to their counterparts in the training datasets. In summary, our study for the first time develops a CNN-based framework for identifying the phage-specific protein sequences with high complexity and low conservation, and this framework will help us find novel phages in metagenomic sequencing data. The DeephageTP is available at https://github.com/chuym726/DeephageTP.

## Introduction

Bacteriophages (phages) are the most abundant and diverse biological entity on the Earth. With the advent of the high-throughput sequencing technologies, amount of microbial metagenomic sequencing data is growing exponentially. Phages are widely present in various environments and thus the phage-originated sequences are present in the metagenomic sequencing data. Particularly, it is estimated that around 17% sequences of the human gut metagenomes are derived from phage genomes (Ogilvie et al., 2013). However, it remains a challenge to identify phage sequences from the metagenomic data due to the following aspects: (a) the phage genomes are highly diverse and lack universal marker genes akin to 16S rRNA genes of bacteria or archaea (Edwards & Rohwer, 2005); (b) most bacterial viruses remain uncultured as their hosts are unknown (Pedulla et al., 2003). These limit our investigations into a complex microbial community to understand the roles of phages in complex ecosystems.

To identify the phage sequences from the complex microbial sequencing data, one common practice is to examine the phage-specific genes encoded by the metagenomic sequences. Thus, if a given predicted protein sequence shows significantly high similarity with the specific proteins of known phages, the metagenomic sequence encoding this protein could be selected as the candidate of phage sequence. In this regard, several alignment-based methods have been developed and extensively utilized, such as BLAST, PSI-BLAST (Altschul et al., 1997), HMM (Hidden Markov Models) (Finn, Clements & Eddy, 2011), etc. Nonetheless, these alignment-based methods mainly rely on reference sequences of phage, usually leading to the failure of detecting the novel phages that encode proteins with poor similarity to those of the reference phages.

Recently, many alignment-free approaches have been developed for identifying and annotating the proteins. Specifically, they typically convert each sequence into a feature vector, and then, the computational prediction of the sequence is implemented based on the corresponding feature vector. For instance, several machine learning-based methods (Seguritan et al., 2012; Feng et al., 2013; Ding et al., 2014; Zhang et al., 2015; Galiez et al., 2016; Manavalan, Shin & Lee, 2018; Pan et al., 2018; Tan et al., 2018) utilize the amino acid frequency as the main predictive features of the sequences to identify phage virion proteins, including VIRALpro (Galiez et al., 2016), PVP-SVM (Manavalan, Shin & Lee, 2018), and iVIREONS (Seguritan et al., 2012). One of the main problems of these methods is that, the number of the possible combinations of amino acids (*i.e.*, 20^*k*^, *k* is the length of amino acid sequence) is extremely high. This makes it difficult for the dimension of the feature vector to tolerate the increase in the value of *k*. Therefore, these methods usually set the value of *k* to be less than four. This, in turn, will lead to the loss of the information, and thus, the prediction performance of the methods could be significantly impaired. Among alignment-free methods, some deep-learning based models show a promising performance, such as DeepFam (Seo et al., 2018), DEEPre (Li et al., 2018), mlDEEPre (Zou et al., 2019), DeepFunc (Zhang et al., 2019), and DeepGo (Kulmanov, Khan & Hoehndorf, 2018). Most recently, DeepCapTail (Abid & Zhang, 2018) has been proposed for predicting capsid and tail proteins of the phage using deep neural network. It suffers from the same limitation of utilizing the amino acid frequency as the predictive features of the sequences. Moreover, it has not been applied to the real metagenomic dataset for examining the actual effect.

To overcome these limitations, in this study, we developed a framework DeephageTP (Deep learning-based phage Terminase and Portal proteins identification) for identifying three specific proteins of *Caudovirales* phages, *i.e.,* TerL (large terminase subunit), Portal, and TerS (small terminase subunit). These three proteins are the key components of the molecular machine of *Caudovirales* phage (*i.e.,* portal (Portal protein), motor (terminase large subunit protein, TerL) and regulator (terminase small subunit protein, TerS)) and this machine plays a crucial role in packaging the phage genome into the phage head capsid. The proposed framework was applied on three real metagenomic datasets to assess its efficacy. Our proposed framework provides the potential opportunity to recognize the new phage at a large scale from metagenomic datasets.

## Materials & Methods

### Datasets

**Figure 1:**
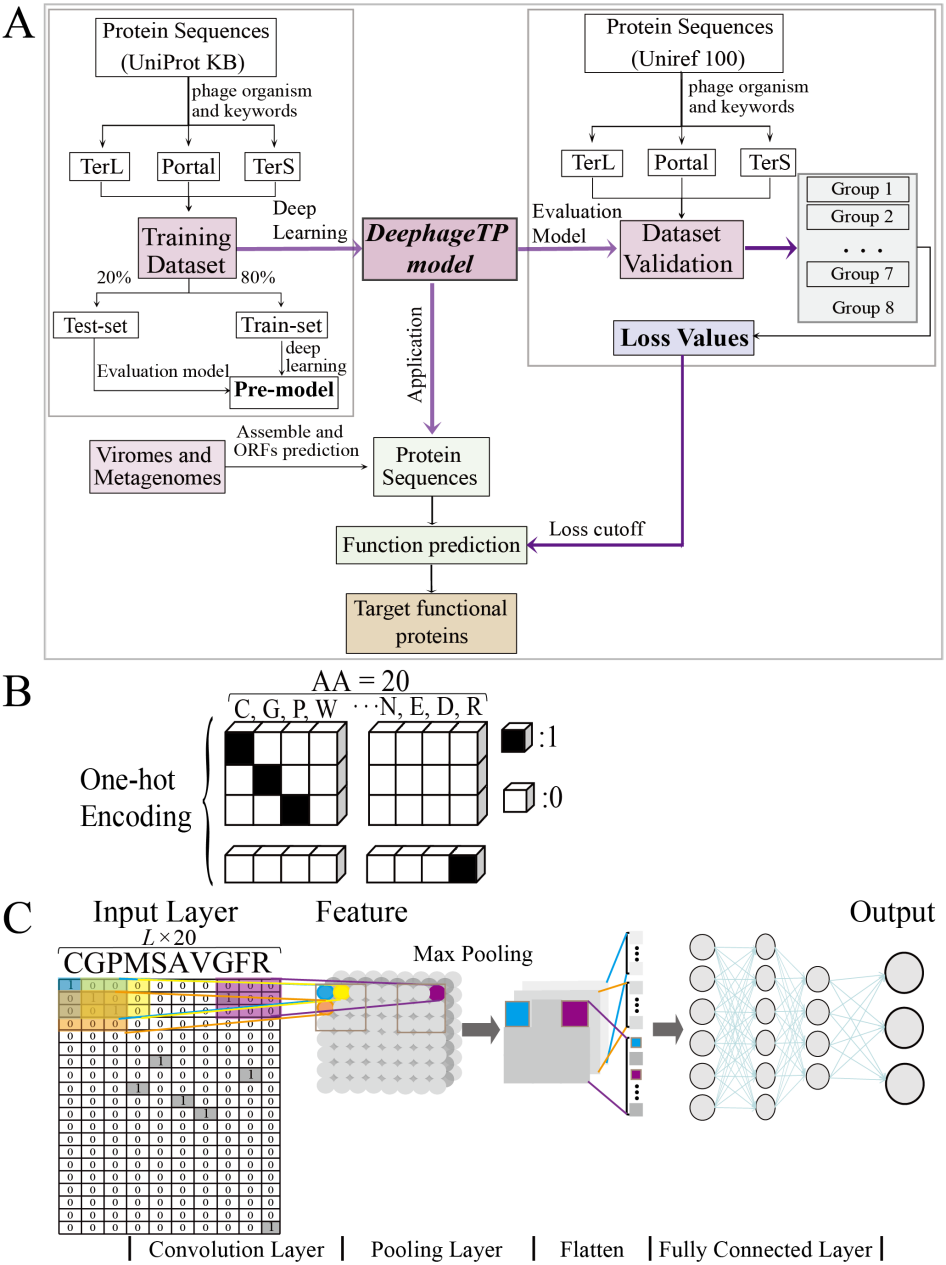
Overview of the framework DeephageTP. (A) The workflow of the proposed DeephageTP framework. The CNN-based model was firstly implemented on the training dataset. Then, the trained model was applied on the mock metagenomic dataset and the cutoff loss value of each category of interest was determined. Finally, the trained model was applied to the real metagenomic datasets for validating the performance of our framework. (B) One-hot encoding for protein sequence. Each amino acid is represented as a one-hot vector. (C) The process of the CNN-based model. Firstly, the amino acid sequence was encoded to ‘one-hot’ and then input into the ‘Input layer’. Then ‘maximum pooling’ was used to extract the features of protein sequence. Finally, the ‘fully connected layer’ was connected with the ‘output layer’, and the final classification results are output.

The collection of the phage protein sequences is obtained from the database: UniportKB (http://www.uniprot.org). Because the proteins including portal, TerL, and TerS, are crucial to the phage (Gao, Zhang & Rao, 2016; Hilbert et al., 2017), thus genomes (or genome fragments) encoding these three functional genes from metagenomic data can be identified as the candidates of *Caudviridae* phages. Without loss of generality, we focus on these proteins in this study. The steps of constructing the training dataset are described as follows (Fig. 1A): (i) according to the taxonomy in the UniProt database, all proteins in archaea, bacteria, and viruses were obtained from the database; (ii) the protein sequences were searched by the keywords (*i.e.,* portal, large terminase subunit, and small terminase subunit), and the noise sequences with the uncertain keywords (*e.g.*, hypothetical, possible, like, predicted) were removed to ensure that the selected protein sequences in the three categories are veracious; (iii) the remaining sequences without the keywords of interest (portal, large terminase subunit and, small terminase subunit) were labeled as the ‘others’ category. The TerL, TerS and portal proteins were selected from UniportKB ‘TrEMBL’ dataset; and the ‘other’ proteins were from UniportKB ‘Swiss-Prot’ dataset with priority deleted TerL, TerS and portal sequences. However, the size of the ‘others’ category is more than 75 times larger than that of the three categories. To relieve the class-imbalance problem brought by this situation, we randomly selected 20,000 protein sequences from the remaining sequences and labeled as the “others” category; (iv) to further guarantee that the sequences from the database with the three categories are veracious, we calculated length distribution of these sequences (see Fig. S1), then manually checked the sequences with the abnormal length (<5% and >95%) using Blastp (https://blast.ncbi.nlm.nih.gov/Blast.cgi) against NCBI NR database, and the sequences that do not hit to the targeted references in the NCBI NR database were filtered out (almost all the sequences with abnormal length) and labeled as the ‘others’ category. The training dataset is summarized in Table 1.

To test the proposed model, we also constructed a mock metagenomic dataset by collecting the protein sequences from another database: UniRef100 (https://www.uniprot.org/uniref/). The collection process for the mock metagenomic dataset is similar to that of the training dataset. It should be noted that the two databases (*i.e.,* UNIPROTKB and UniRef100) overlap in some sequences, and thus we manually deleted the sequences that exist in the training dataset from the mock dataset. To this end, the mock dataset can be regarded as an independent dataset from the training dataset. In particular, to investigate the prediction performance of the model on the test data with different size, we generated seven groups of data (*i.e.,* Group one to Group seven) from the original mock dataset (*i.e.,* Group eight), where except for the three category proteins, the samples from the “others” category were randomly selected from the Group eight. Here, since we mainly focus on the impact of different data sizes on the performance of the proposed model in identifying the three category proteins, the samples of the three category proteins were kept the same for eight groups of the data. Table 2 describes the details of the datasets used for test analysis.

**Table 1:** The numbers of proteins of each category in the training dataset. A total of 80% train-set and 20% test-set are used for feasibility analysis, and the training dataset (including train-set and test-set) are used for training the proposed model.

Protein categories	Training dataset	
	80% train-set	20% test-set
**TerL**	2093	524
** Portal**	2607	653
**TerS**	1202	301
** others**	16163	4042

To assess the performance of the proposed model on the real metagenomic dataset, we collected the virome dataset from the wastewater (accession number in NCBI: SRR5192446) and two virome datasets from the human gut (accession number in NCBI: SRR7892426 and ERR2868024) (Moreno-Gallego et al., 2019; Yinda et al., 2019). As the data of these datasets are sequencing reads, we first assembled them using SPAdes 3.11.1 (Bankevich et al., 2012) and applied Prodigal (Hyatt et al., 2010) for gene calling with the default parameters. As a result, we obtained 366,146 (SRR5192446), 110,129 (SRR7892426), and 27,157 (ERR2868024) protein sequences for these datasets, respectively.

**Table 2:** The numbers of proteins of each category in the mimic metagenomic dataset and the seven testing groups.

Testing datasets	TerL	Portal	TerS	Others
**Group 1**	14437	41398	5918	30000
**Group 2**	14437	41398	5918	50000
**Group 3**	14437	41398	5918	70000
**Group 4**	14437	41398	5918	90000
**Group 5**	14437	41398	5918	110000
**Group 6**	14437	41398	5918	130000
**Group 7**	14437	41398	5918	150000
**Group 8**	14437	41398	5918	476685

### Protein sequence encoding

To tackle the protein sequence data with the proposed model, we firstly formulated an image-like scheme to encode each protein sequence (Fig. 1B). Specifically, each of the 20 amino acids is encoded as a one-hot vector of 20 dimensions (*i.e.,* one-dimension value is one and others are 0, shown in Fig. 1B) (LeCun, Bengio & Hinton, 2015). Based on this, a protein sequence with *L* length (*i.e.,* the number of amino acid residues) could be encoded as a *L* × 20 matrix *X*.

As the lengths of the protein sequences typically varied, and the input data are required to be the same size for the model, we fixed *len_w* (the maximum length of the sequence for modeling) equal to 900 according to the length distribution of the three category proteins due to that almost all lengths of these proteins are less than 900 (Fig. S1). In addition, the minimum loss value and maximum accuracy were obtained with five-fold cross validation on the training dataset with 900 amino acids (Table S1). Specifically, if the length of a given sequence is longer than *len_w*, the excess part of the sequence would be abandoned; else, the insufficient part of the sequence would be filled with multiple ‘-’. Each ‘-’ is encoded as a zero vector of 20 dimensions. Therefore, each protein sequence could be encoded as a *len*_*w* × 20 matrix. These matrixes can be used as the input data for the proposed model.

### The CNN-based deep learning model

The framework DeephageTP is developed based on CNN. The CNN comprises a convolutional layer, a max-pooling layer, two fully connected layers as well as the input and output layers. The dropout technique (Srivastava et al., 2014), which avoids overfitting *via* randomly removing the units during training at a fixed rate (*i.e.,* 0.1 in our experiments), is applied on the pooling layer and the first fully connected layer in the model. One of the most common activation function *ReLu* (LeCun, Bengio & Hinton, 2015) is used on the convolutional layer and the first connected layer, while the output layer utilizes *SoftMax* (Zang & Zhang, 2011) as the activation function to compute the probability of the protein sequence against the category. The CNN model is shown in Fig. 1C.

It is worth noting that there are many hyperparameters in the model such as the number of the convolution kernels, the number of units in fully connected layers, the dropout rate, the learning rate, etc. For which, it is difficult to obtain the optimal values of these parameters. To this end, for most of these parameters, in the process of modeling, we used the default settings that are widely applied in practice (LeCun, Bengio & Hinton, 2015), while the remaining parameters were tuned according to the averaged prediction performance of the proposed model on the training dataset using the five-fold cross-validation. The structure of the CNN was determined by examining four main hyper-parameters (Zeng et al., 2016), including the length size of protein sequences, kernel size of the filter, number of filters for each kernel size, and the number of neurons in fully connected layer (Seo et al., 2018). These parameters were selected according to our experiences and the references (Savojardo et al., 2018; Arango-Argoty et al., 2018). 20 proteinogenic amino acids were classifid into seven groups (seven-letter reduced sequence alphabets) according to their dipole moments and side-chain volume: {A,G,V}, {I,L,F,P}, {Y,M,T,S}, {H,N,Q,W}, {R,K}, {D,E} and {C}(Suresh et al., 2015). The kernel size of the filter was set to 7 × 1 in the light of the previous studies (Suresh et al., 2015; Yi et al., 2019); we examined the values of 800, 900, and 1,000 for the length of sequences based on the distribution of the length; we also examined the values of 30, 50, 70 and 90 for the number of filters, as well as the values of 50, 100, 150 and 200 for the number of neurons in the fully connected layer. Specifically, we evaluated the performance of the model with different values of the parameters using five-fold cross-validation on the training dataset, and the results are shown in Tables S1–S3. Finally, we set the length size to 900, the number of filters to 50, and the number of the neurons in the fully connected layer to 100.

The architecture of the DeephageTP framework is implemented using the Python Keras package (https://keras.io), a widely used, highly modular deep learning library. The DeephageTP is available at https://github.com/chuym726/DeephageTP.

### Evaluation metrics

To evaluate the performance of the proposed model, four widely used metrics, *i.e., Accuracy*, *Precision*, *Recall*, and *F1-score* were applied in this study and defined as:

\begin{eqnarray*}Accuracy= \frac{TP+TN}{TP+FP+TN+FN} , \end{eqnarray*}

\begin{eqnarray*}Precision= \frac{TP}{TP+FP} , \end{eqnarray*}

\begin{eqnarray*}Recall= \frac{TP}{TP+FN} , \end{eqnarray*}

(1) \begin{eqnarray*}F1-score= \frac{2\ast Precision\ast Recall}{Precision+Recall} ,\end{eqnarray*}

where TP denotes true positives (*i.e.,* a protein sequence from one of the categories is predicted correctly as the category), TN (true negatives, a protein sequence comes from other categories of interest is predicted correctly as the other category), FN (false negatives, a protein sequence comes from the category of interest is wrongly predicted as the other category), and FP (false positives, a protein sequence comes from a different category is wrongly predicted as the category of interest). *Accuracy* reflects the overall prediction quality of the model. *Precision* focuses on measuring how accurate the categories of the phage protein sequences predicted by the model are, while Recall measures the proportions of the phage protein sequences that are correctly identified by the model. And *F1-score* is the harmonic mean of *Precision* and *Recall*.

### Loss value computation

To determine the appropriate cutoff loss values for the three protein categories, we considered the loss value of each sequence. The loss value is calculated according to the loss function used in the proposed model. The loss value is a score criterion that reflects the difference between the real category of the sequence and the predicted category of the sequence. The smaller the loss value is, the smaller the difference is. Specifically, the widely applied cross-entropy function (LeCun, Bengio & Hinton, 2015) was employed in this study and defined as follows:

(2) \begin{eqnarray*}L=-\sum _{k=0}^{K-1}{y}_{k}\log \nolimits {p}_{k}\end{eqnarray*}

where *y*_*k*_ is the value of the real label of the sequence on the *k*-th dimension, and p_k_ is the corresponding value on the *k*-th dimension that is predicted by the model. For most deep learning models, the category label is typically encoded as a one-hot vector (*i.e.,* one-dimension value is one and others are 0) with k dimensions, and the predicted value for each dimension is calculated *via* the *SoftMax* function.

Additionally, in general, the averaged loss value for all sequences is used for evaluating the performance that the model fits the dataset. However, in this study, we utilized the loss value for each sequence to determine the cutoff values. The main reason is that, if a sequence is predicted as one category by the trained model with a very small loss value, it means that the sequence is much the same as the sequences within the category, and the smaller the value is, the more likely it would be. On the other hand, if the loss value is relatively large, although the sequence is predicted as the category by the model, it would likely be false positives. To this end, according to the distribution of the loss values with the same category, the bounds that distinguishing TP and FP will be determined.

### DeephageTP application on real metagenomic datasets

To assess the performance of the proposed framework on real metagenomic data in identifying phage sequences, we applied the framework on the three real metagenomic data. Specifically, the proteins of the three categories predicted by the model were selected and then filtered with the cutoff loss values determined above. Finally, we manually checked the DeephageTP-identified protein sequences using DIAMOND (Blastp model) (e-value: 1e−10) against NCBI NR database. According to the results, the identified sequences can be divided into four groups: (a) true-positive: the sequence has Blastp hits in the NCBI NR database within the same category as DeephageTP predicted (as long as one hit in the result list of Blastp against NCBI NR database is annotated to the category of interest); (b) phage-related: at least one of the protein sequences carried by the contig where the identified protein gene is located has hit to other phage-related proteins (as long as one is annotated to phage-related protein in the result list of Blastp); (c) Unknown, the sequences don’t have hits or the hits are annotated as hypothetical protein; (d) Other function, the sequences have hits annotated as other functional proteins that likely are derived from bacterial genomes (none of the hits in the result list of Blastp are annotated as phage-related proteins).

### Alignment-based methods for comparison

Two major alignment-based methods, Hidden Markov Model (HMM) (Eddy, 2011) and Basic Local Alignment Search Tool (BLAST) (Altschul et al., 1997) were used to annotate the protein sequences and the results were compared with those of our method in the experiments. Specifically, multiple sequence alignments were generated firstly using MUSCLE v3.8 (Edgar, 2004) for three phage-specific proteins in the training dataset. Then, the HMM algorithm was constructed using HMMER v3.1 (http://hmmer.org/). For each sequence alignment, we built HMM of each protein category *via* hmmbuild, where the models were compressed into a single database indexed with hmmpress. For each test protein sequence, hmmscan scored the significance that the sequence matched to the categories of interest with *E*-value, and the category with the most probable (*i.e.,* the one with the smallest *E*-value) was chosen as the output. In some cases, the *E*-value could not be yielded from the constructed models, where the sequences were discarded in our experiment. The two software (*i.e.,* MUSCLE v3.8 and HMMER v3.1) were set with default parameters for implementation. For the BLAST method, we used the software DIAMOND (Buchfink, Xie & Huson, 2015) to find the most similar sequences in the database (created with the proteins in our training dataset) for a test protein sequence and assign its category to the test sequences. The cutoff e-value of the DIAMOND program was set 1e−10 in our experiments.

## Results

### Prediction performance of the CNN-based model on the training dataset

**Figure 2:**
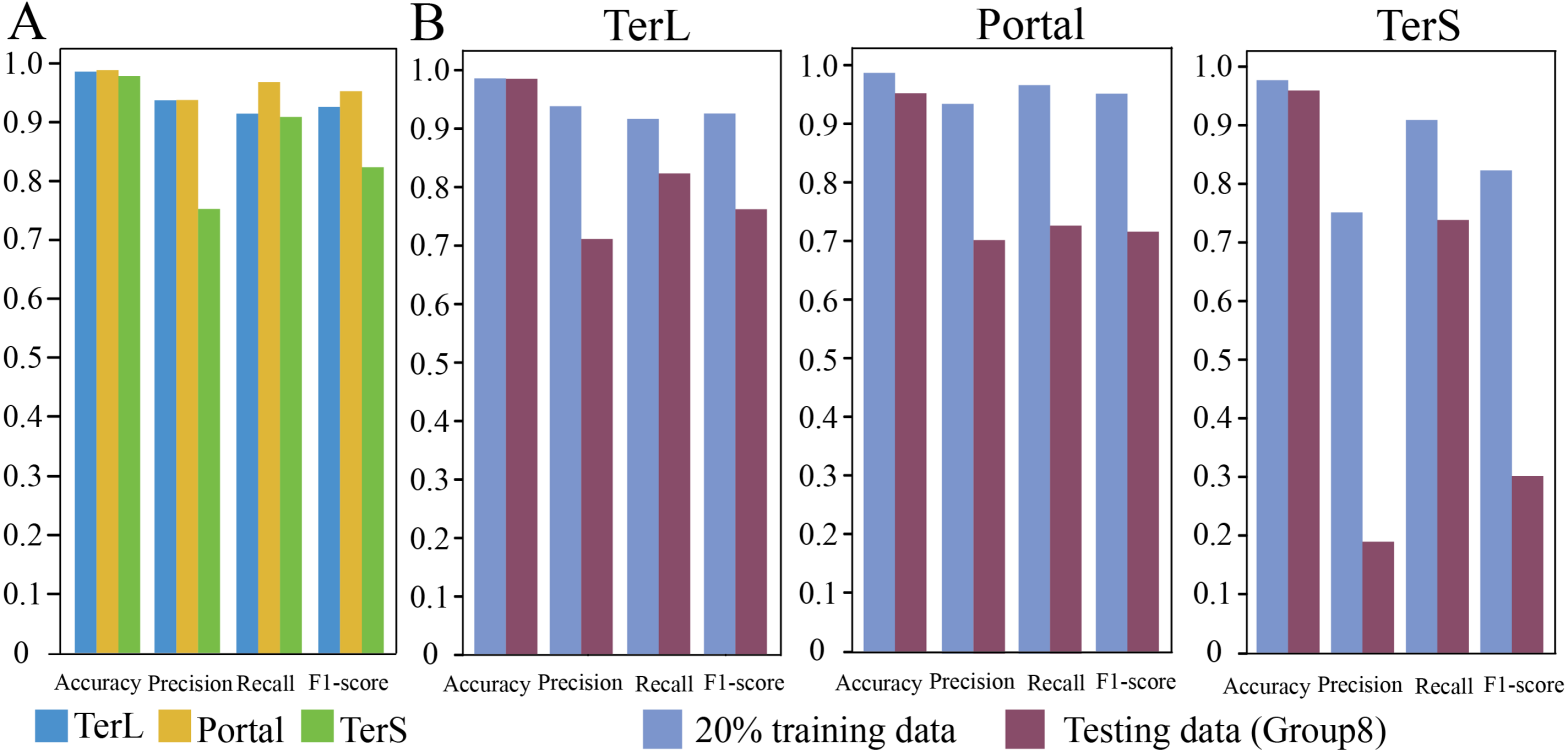
Prediction performance of the CNN-based model. (A) Performance of the model on the training data. The model was trained on the train-set (80% training data), and the prediction performance was evaluated on the test-set (20% training data) with four metrics (*i.e., Accuracy, Precision, Re call and, F1-score*) for the three phage proteins, respectively. (B) Comparison of the prediction performance of the model on the test set of the training dataset and the mock metagenomic dataset. The prediction performances for two datasets (purple: the test set of the training dataset, green: the mock dataset) were evaluated with four metrics (*i.e., Accuracy, Precision, Re call and, F1-score*) for the three phage proteins, respectively.

In training dataset, 80% of sequences of each category were randomly selected for training the proposed model, while the remaining 20% were used for testing. The results are shown in Fig. 2A. As it can be observed that, in general, the proposed model show relatively high prediction performance on the dataset; over 97% accuracy can be achieved for the three protein categories (Portal: 98.8%, TerL: 98.6%, TerS: 97.8%), respectively. The best prediction performance was obtained on Portal protein in terms of *Precision*, *Recall*, and *F1-score* (93.88%, 96.94%, and 95.33%, respectively). The relatively high prediction performance achieved for TerL (*Precision*: 93.75%, *Recall*: 91.60%, *F1-score*: 92.66%, respectively). The prediction of TerS generated the lowest performance (*Precision*: 75.28%, *Recall*: 91.03%, *F1-score*: 82.41%, respectively), especially for *Precision*, suggesting that nearly a quarter of TerS sequences could not be correctly identified by the model.

### Prediction performance of CNN-based model on mock metagenomic dataset

To further assess the proposed model, we prepared an independent mock metagenomic dataset from another database (UniRef100 database). We applied the trained model on mock dataset (Group eight) (Table 2). As shown in Fig. 2B, we found that, except for *Accuracy*, the prediction performances in terms of other metrics significantly became worse (TerL71.1%, Portal 70.5%, TerS 19.1% (*Precision*); TerL 82.3%, Portal 73.0%, TerS 73.9% (*Recall*); TerL 76.3%, Portal 71.7%, TerS 30.3% (*F1-score*)) for the three proteins when compared with those on the training dataset. This is likely because, in mock dataset, the number of sequences from the “others” category is much larger than that of sequences from the category of interest (*i.e.,* class imbalance).

**Figure 3:**
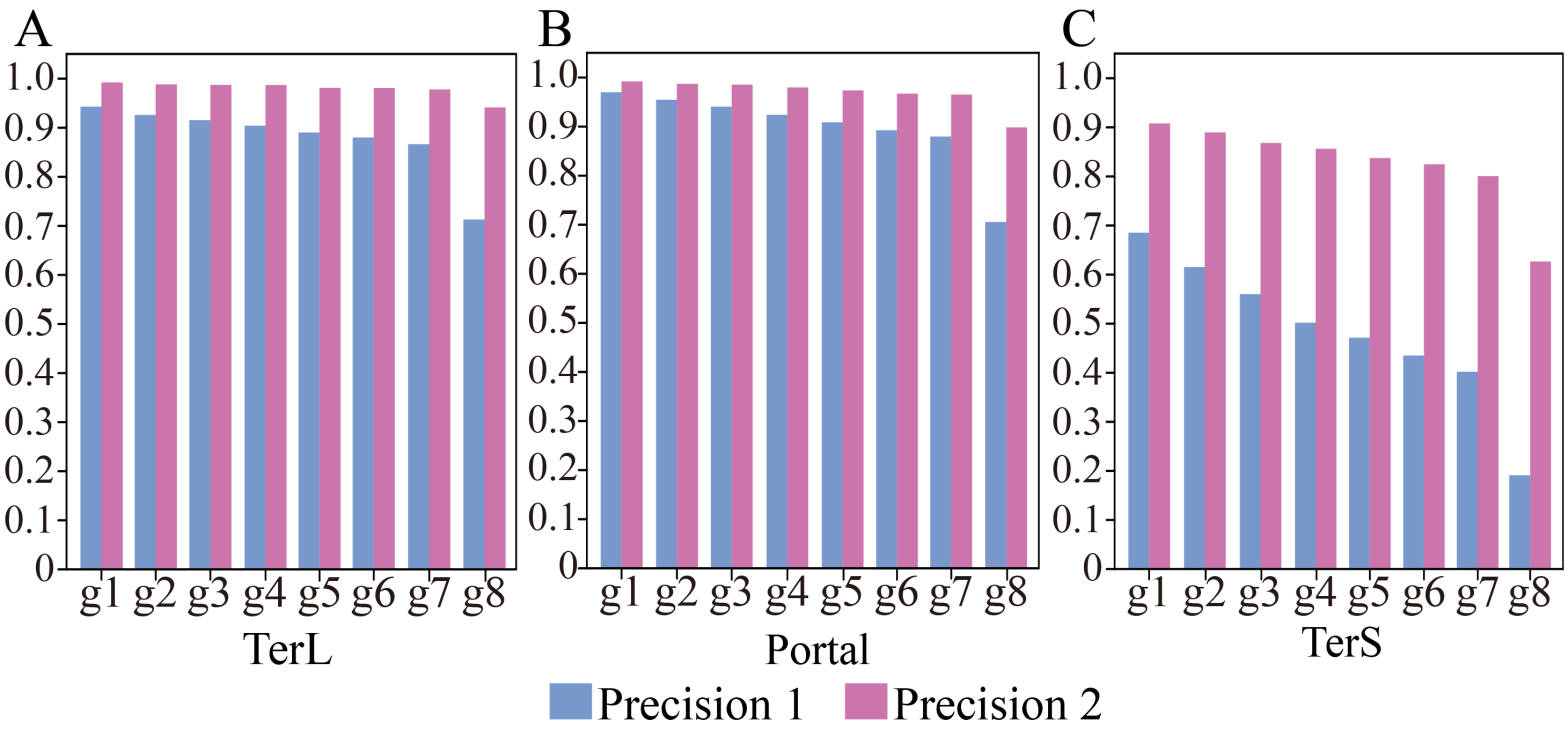
(A–C) Performances of the model with and without cutoff loss values on the mock metagenomics dataset. The performance was evaluated in terms of *Precision* (Precision 1, without cutoff loss values; Precision 2, with cutoff loss values). Seven groups (Group 1-7) with different sizes were generated from the mock metagenomic dataset.

Thus, we further applied the trained model on the seven groups of the data, respectively, to assess the impact of such class imbalance on the prediction performance of the model in identifying the three phage-specific protein sequences. The mock dataset was divided into seven groups with different sizes (Table 2). The results are shown in Fig. 3 and Table S4. Compared with the results on Group 1, *Precision* and *F1-score* values for the three proteins decreased significantly (by TerL 1.6%–23.2%, Portal 1.5%–26.4%, TerS 7.0%–49.5% (*Precision*); TerL 0.7%–11.6%, Portal 0.6%–11.6%, TerS 15.6%–52.4% (*F1-score*)) with the dataset size increasing, while the *Recall* values remain unchanged. This indicates that the number of true-positive sequences from the categories of interest was not impacted by the size of the dataset. However, with the testing dataset size increasing (Table 2), more and more sequences from the “others” category were wrongly predicted as the category of interest by the model (*i.e.,* the FP value becomes larger). Since the *Recall* values are the same for all testing datasets, the *F1-score* values are only affected by the *Precision* values and the trend of the *F1-score* values are similar to that of the *Precision* values. Therefore, we focus on the prediction performance in terms of *Precision* in the following experiments.

**Figure 4:**
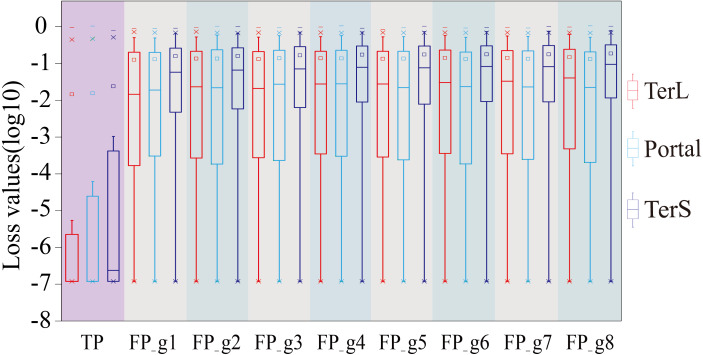
The loss value distributions of TP and FP for the three phage proteins on the mock metagenomic dataset. Group 1-7 datasets were generated from the mock metagenomic dataset (group eight). The loss value distributions of TP (all are the same for eight groups) and FP were calculated on the eight groups, respectively, for the three phage proteins. TP: true positive; FP: false positive. g1-g8: Group1-Group8.

**Figure 5:**
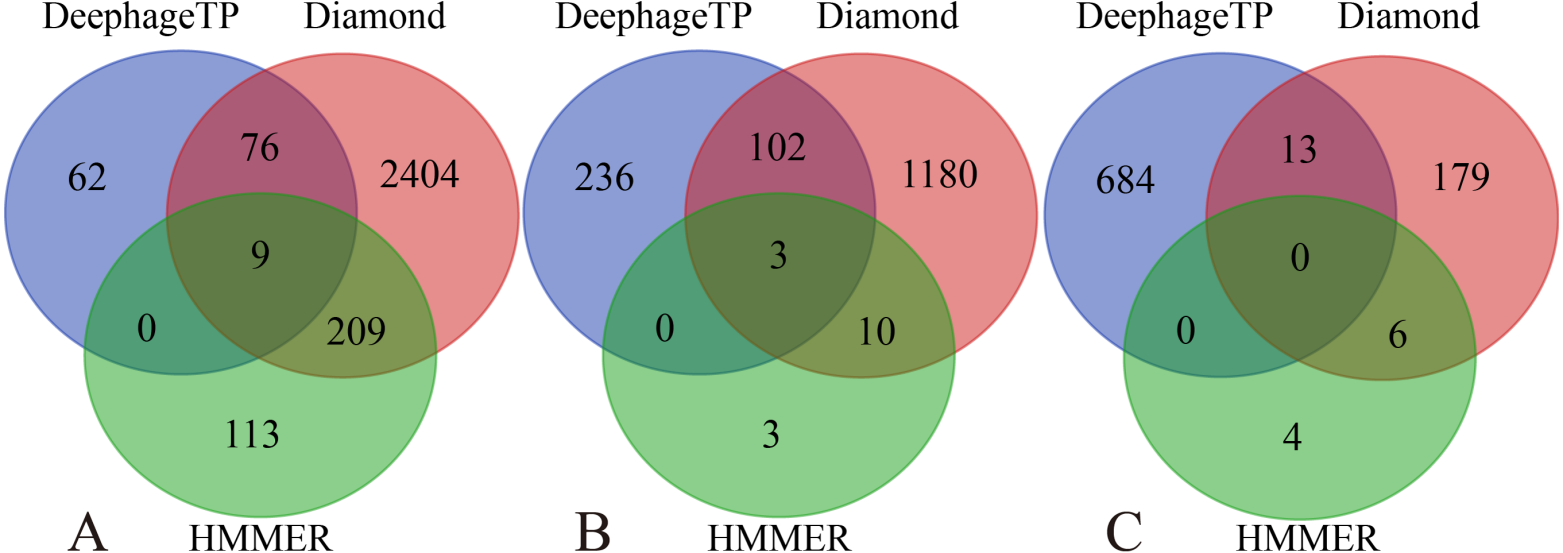
Venn diagrams of the prediction results of three methods (*i.e.,* DeephageTP, Diamond and HMMER) on the metagenomic dataset (SRR5192446). (A) TerL; (B) Portal; (C) TerS.

Therefore, we further employed a new strategy to improve the prediction performance of the model in terms of *Precision* by introducing the appropriate cutoff loss values for each category of interest. Specifically, we first calculated the distributions of the loss values of the sequences correctly identified (*i.e.,* TP) and the sequences wrongly predicted as the categories of interest (*i.e.,* FP) by the trained model for the three protein categories using the eight groups of the mock metagenomic dataset, respectively (Table 2); based on this, the loss value for a given category that may distinguish the TP and FP for most sequences would be chosen as the corresponding cutoff values. It should be noted that, as mentioned above, the TP values of the three protein categories are the same in the eight groups of the mock metagenomic datasets, so the distributions of the corresponding loss values were shown in Fig. 4. Since the majority of the loss values of TP sequences are relatively low (loss values (log10, the same below): TerL <−5.2, Portal <−4.2, TerS <−2.9) while those of FP sequences are relatively high (loss values: TerL >−4.0, Portal >−3.6, TerS >−2.5) for the three proteins on all groups, thus, the corresponding cutoff values of three phage proteins for distinguishing TP and FP could be selected with relative ease. Because the distributions of the loss values for three proteins are different, thus it is essential to set the appropriate cutoff values for each of them. In this study, we chose the values at the top of the boxplots of the three TP protein sequences in Fig. 5 (*i.e.,* TerL: −5.2, Portal: −4.2, TerS: −2.9) as the cutoff values for the three categories, respectively. With these cutoff values, we can observe most TP sequences (>99%) in the mock metagenomic dataset (group eight) were identified correctly. A stricter cutoff value could also be selected according to the practical necessity and the consideration of the balance between false-positive rate and false-negative rate.

With the determined cutoff loss values, we reassessed the prediction performance of the model on the eight groups of the mock metagenomic dataset. Specifically, the sequences that originally were predicted as the category of interest but with the loss value larger than the corresponding cutoff value would be predicted as the “others” category instead. As shown in Fig. 3, Table S4 and Table S5, compared with the results obtained without using the cutoff values, the performance of the new strategy shows remarkable improvements in terms of *Precision* (improved by TerL 4.9–22.8%, Portal 2.2–19.3%, TerS 22.2–43.5%) for the eight groups, although the prediction performance in terms of *Recall* somewhat decreases. Moreover, compared to the result of group one, with the increasing sizes of the groups, the *Precision* values reduced by TerL 0.3−5.3%, Portal 0.5−9.4%, TerS 1.5–28.1% for the three proteins, which were much less than those of without using the cutoff strategy. In particular, the *Precision* values for TerL and Portal can still reach 94% and 90% respectively, even on the mock dataset (*i.e.,* Group eight) that is 20 times larger than the training dataset. This result demonstrates that, by introducing the cutoff values, the effect of the excessive size of the testing data would be reduced to a relatively small degree.

It worth noting that, in all these experiments, the model showed much worse prediction performance in identifying TerS sequences than the other two proteins (Fig. 3, Tables S4, S5), although the introduction of cutoff loss value can significantly improve the performance of the model in terms of *Precision* (21–42%). This is likely because the number of TerS used for training is much less than those of the other two proteins.

### Application of framework DeephageTP on real metagenomic datasets

We applied the framework on the three real metagenomic sequencing datasets with the corresponding cutoff loss values (TerL: −5.2, Portal: −4.2, TerS: −2.9) to identify the phage-derived sequences. Finally, 1,185 out of 366,146 protein sequences (TerL: 147, Portal: 341, TerS: 697) were identified from the dataset (SRR5192446) by our method, 42 out of 27,157 protein sequences (TerL: nine, Portal: 15, TerS: 18) from ERR2868024 and 127 out of 110,129 protein sequences (TerL: 16, Portal: 23, TerS: 88) from SRR7892426. The dataset (SRR5192446) has a higher number of identified sequences of interest than the other two. This result is in line with those of two alignment-based methods (*i.e.,* DIAMOND and HMMER). It can be observed that the total numbers of the three phage proteins predicted from the sample (SRR5192446) by the two alignment-based methods are 4,200 (DIAMOND) and 357 (HMMER) respectively, much higher than those from the other two datasets (ERR2868024, and SRR7892426). This is likely because the sample (SRR5192446) was collected from the environment of waste-water and the majority of the sequences in the training dataset were collected using environmental microbes. Among the protein sequences identified by the three methods from the dataset of waste-water (SRR5192446), a few sequences (TerL 85, Portal 105, TerS 13) are shared by DeephageTP, and DIAMOND, some (TerL 9, Portal 3, TerS 0) shared by DeephageTP and HMMER, but very few can be identified by the three methods simultaneously (Fig. 5), suggesting that the phage-specific protein sequences identified by DeephageTP are different from those of alignment-base methods, and these protein sequences are likely derived from novel phage genomes in the metagenomes. This case is similar to those of the other two datasets from human gut samples (Fig. S2).

**Figure 6:**
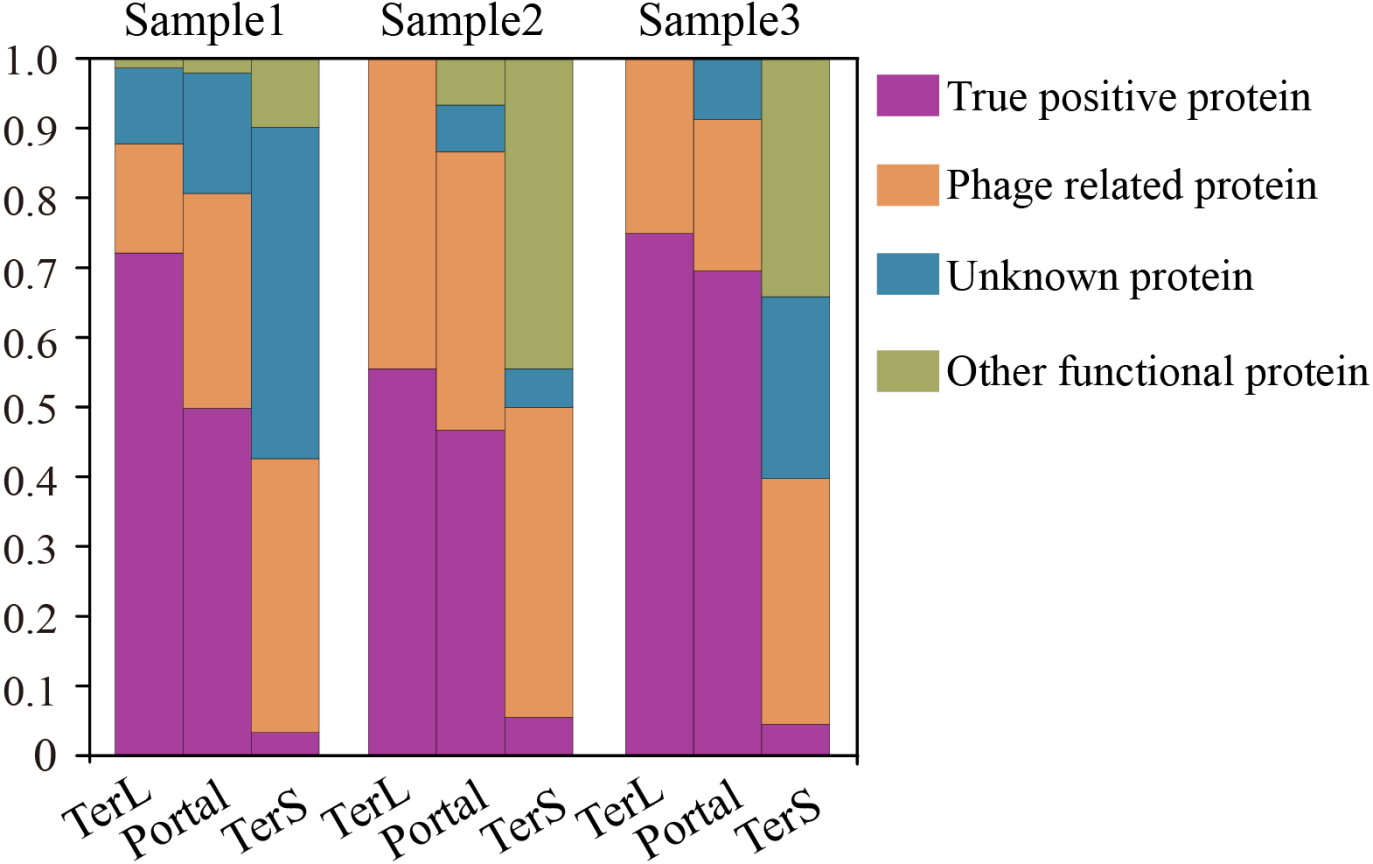
Verification of the three phage proteins identified by DeephageTP from the metagenome datasets. (Sample1: SRR5192446, Sample2: SRR7892426 and Sample3: ERR2868024). (A) true positive: the sequence has Blastp hits in the NCBI nr database within the same category as DeephageTP predicted (as long as one hit in the result list of Blastp against NCBI nr database is annotated to the category of interest); (B) phage-related: at least one of the protein sequences carried by the contig where the identified protein gene is located has hits to other phage-related proteins (as long as one is annotated to phage-related protein in the result list of Blastp); (C) Unknown, the sequences don’t have any hits or the hits are annotated as hypothetical protein; (D) Other functional, the sequences have hits annotated as other functional proteins that likely are derived from bacterial genomes (none of the hits in the result list of Blastp are annotated as phage-related proteins).

To further confirm the sequences identified by DeephageTP, we manually checked the protein sequences using Blastp (E-value:1e−10) against the NCBI nr database. As shown in Fig. 6, the results demonstrate that, again, few DeephageTP-identified TerS sequences were verified in the NCBI nr database as true positive (SRR5192446: 22 (3.16%), ERR2868024: 1 (5.56%), SRR7892426: 4 (4.55%)). However, in regard to TerL and Portal, a large fraction of the protein sequences were confirmed as the true positive (SRR5192446: TerL 105 (71.4%), Portal 172 (50.4%); ERR2868024: TerL 5 (55.6%), Portal 7 (46.7%); SRR7892426: TerL 12 (75%), Portal 16 (69.6%)).We further examined the whole contigs that carry the remaining identified protein sequences. According to the hits of each protein carried by the contigs, only a small number of identified proteins belong to other functional proteins likely encoded by bacterial genomes (SRR5192446: TerL 6 (4.1%), Portal 7 (2.1%); ERR2868024: TerL 0 (0%), Portal 1 (6.7%); SRR7892426: TerL 0 (0%), Portal 0 (0%)). Note that, a considerable proportion of the identified proteins are encoded by phage-derived contigs (SRR5192446: TerL 20 (13.6%) Portal 103 (30.2%) TerS 243 (34.9%), ERR2868024: TerL 3 (33.3%) Portal 6 (40%) TerS 8 (44.4%), SRR7892426: TerL 4 (25%) Portal 5 (21.7%) TerS 31 (35.2%)) and quite a part of the predicted proteins belong to unknown proteins (SRR5192446: TerL 16 (10.9%), Portal 59 (17.3%) TerS 351 (50.4%), ERR2868024: TerL 1 (11.1%) Portal 1 (6.7%) TerS 0 (0%), SRR7892426: TerL 0 (18.75%) Portal 2 (8.7%) TerS 22 (25%)). Most of these proteins have low identities (<30%) (Table S6) to the hits in the NCBI nr database, suggesting some of them are likely novel TerL encoded by novel phages, which needs further investigations. Among the protein sequences identified by DeephageTP and confirmed as the true positive, a number of proteins were not determined by the other two alignment-based methods (Table S6). For example, 10.2% (15/147) TerLs and 37.8% (65/172) Portals were only detected by DeephageTP in sample SRR5192446. This indicates that DeephageTP is capable of recognizing novel phage genes of interest. These novel genes are great divergent from their reference ones, and thus, may be ignored by alignment-based methods.

## Discussion

Bacteriophages are present in all kinds of the microbial communities. With conventional sequence-alignment-based methods, the identification of phage sequences from the metagenomic sequencing data remains a challenge due to the great diversity of the phage and the lack of conserved marker genes among all phages. In this paper, we present a CNN-based deep learning framework, DeephageTP, an alignment-free method to identify three tailed-phage-specific proteins, *i.e.,* TerL, Portal, and TerS. In doing so, we can further recognize phage-derived sequences encoding the three proteins from metagenome sequencing data.

We employed the multiclass classification CNN model in this study. In general, the identification of the three proteins can be deemed as three binary classification problems (one-vs-all scheme) or a multiclass classification problem (Sáez et al., 2015). The former divides the original data into two-class subsets and learns a different binary model for each new subset. It may bring more cost of calculation than the latter as it learns multiple different models. We also compared the prediction performances of these two strategies using the training dataset, and the results are shown in Table 3. It can be seen that the two strategies have similar prediction performance to a large extent. Specifically, for TerL, the binary models performed a bit better than the multiclass model (*Accuracy*: 98.82% *vs* 98.58%; *Precision*: 95.45% *vs* 93.75%; *Recall*: 91.98% *vs* 91.60%; *F1-score*: 93.68% *vs* 92.67%). For Portal, the binary models achieved better performance in terms of *Accuracy*, *Precision* and *F1-score* (*Accuracy*: 99.24% *vs* 98.84%; *Precision*: 99.19% *vs* 93.78%; *F1-score*: 96.7% *vs* 95.33%). Meanwhile, the multiclass model obtained better prediction performance in terms of *Accuracy*, *Precision* and *F1-score* (*Accuracy*: 97.83% *vs* 96.96%; *Precision*: 75.28% *vs* 65.80%; *F1-score*: 82.41% *vs* 76.73%) for TerS. Considering the cost of computation, we used the multiclass classification model rather than the binary classification models in this study.

**Table 3:** Comparison of prediction performances of multiclass classification model and binary classification model on the test-set of the training datasest.

Proteins	Multiclass classification				Binary classification			
	Accuracy	Precision	Recall	F1	Accuracy	Precision	Recall	F1
TerL	0.9858	0.9375	0.916	0.9267	**0.9882**	**0.9545**	**0.9198**	**0.9368**
Portal	0.9884	0.9378	**0.9694**	0.9533	**0.9924**	**0.9919**	0.9433	**0.967**
TerS	**0.9783**	**0.7528**	0.9103	**0.8241**	0.9696	0.658	0.9203	0.7673

In a microbial community, viruses generally make up a relatively small fraction of the genome compared to bacteria and fungi. This class imbalance problem can affect the performance of our framework. We applied the trained model on an independent mock metagenomic dataset (20 times larger than the training dataset) and found that the prediction performance in terms of *Precision*, *Recal* l, and *F1-score* decreased remarkably. In the mock dataset, many sequences from the “others” category are different from those in the training dataset, and these sequences are wrongly identified as the category of interest by the trained model (*i.e.,* false-positive problem). This leads to the reduction of *Precision*. Meanwhile, a part of sequences belong to the category of interest are dissimilar to those in the training dataset; thus, they are wrongly predicted as the other category by the trained model (*i.e.,* false-negative problem), resulting in the reduction of *Recall*. The descent degree of *Recall* is less than that of *Precision*, especially for TerS. The reduction of *F1-score* is inevitable as it is the harmonic mean of *Precision* and *Recall*.

To further examine the impact of the data size on the prediction performance of the model, we conducted the experiments on the seven additional groups from the mock metagenomic dataset with different sizes. An interesting finding was that, for the eight groups, the prediction performance in terms of *Recall* was not affected by the data size, while the prediction performance in terms of *Precision* decrease significantly with the increase of the data size. Here, we presented a new way to improve the prediction performance of the proposed model in terms of *Precision* by introducing the cutoff loss values that were determined according to the distribution of the loss values with the category of interest. This strategy can significantly improve the prediction performance of the model in terms of *Precision* for the categories of interest. The larger the size of the testing dataset is, the more significant the improvement of the performance will be. On the other hand, the prediction performance in terms of *Recall* was reduced unavoidably with the strategy compared to the results without the strategy, which means the false-negative rate was raised. Even so, our strategy provides a certain basis for setting a cutoff value of each category that will balance the FP rate and the FN rate.

Our framework demonstrates a remarkable capability to identify new phage protein sequences that have extremely low identities with the known sequences of the training data. In the testing analysis, the framework identified the majority of the three protein sequences (*Recall*, 82.3% TerL, 73.0% Portal and 74.0% TerS, Fig. 3, Table S4) from the mock metagenomic dataset where all the three protein sequences are different from those of the training dataset. Moreover, in the application of the framework on the real metagenomic datasets, the capability of the framework in identifying novel phages also can be observed that our method identified many phage protein sequences that were not detectable by the two alignment-based methods. In this study, we verified the novelty of the DeephageTP-identified sequences by re-annotating them in the NCBI nr database. Experiments including gene express and Transmission Electron Microscope, which are a gold standard for identifying phage particles, are required in further studies (Seguritan et al., 2012).

Nonetheless, we also observed some limitations of the proposed framework in the application. First, only a small number of the phage sequences present in the metagenomic data can be identified by the proposed framework. For example, in sample SRR5192446, 147 (106 true-positives) TerL sequences and 341 (172 true-positives) Portal sequences were identified, as compared with 2,581 and 1,295 by the software DIAMOND, respectively. Similar cases are also observed in the other two human gut metagenomes (Fig. S2). Also, the framework failed to identify the crAss-like phages which are known widely distributed in human gut samples (Table S6) (Guerin et al., 2018). Second, our trained model likely prefers to identify the phages of the environmental microbes instead of those of the human gut microbes. Around 0.029% (106/366,146) of the sequences were identified as true-positive TerL sequences by the framework from the water sample, while only 0.018% (5/27,157) and 0.011% (12/110,129) from the other two human gut samples, respectively. This is likely because the phage sequences recruited by the training dataset are mainly from environmental samples, and in the NCBI nr database, more than 98% of phages are specific to infect the environmental microbes. Third, the performance of the proposed framework in identifying TerS sequences from metagenomic datasets is relatively low in contrast to TerL and Portal sequences. In general, in a given metagenome, the number of TerS is equal to that of TerL, but in all cases in our study, the number of TerSs identified by the framework is around one-fifth of that of TerL. All above limitations of the proposed framework can be attributed to the extremely small number (TerL 2,617, Portal 3,260, TerS 1,503) of the known phage sequences included in the training dataset, compared to the number of phages present in the environmental samples and human gut samples. Therefore, the information extracted from the limited number of the known phages using the framework is insufficient to cover all phage sequences in a given metagenomic sample. Particularly, the low performance of the framework in identifying TerS sequences might be because the number of TerS sequences used for training is much less and the length of the sequences is shorter than those of the other two proteins, and the information provided by the TerS sequences in the training dataset would be insufficient to identify the different TerS sequences in the metagenomic datasets. The shorter the sequence is, the less information is provided to the framework. Thus, to optimize our proposed framework in further study, we will select the appropriate marker sequences with a longer length and include more sequences into the training dataset.

## Conclusions

Here, we devised and optimized a CNN-based deep learning framework for identifying the three phage proteins from complex metagenomic sequencing datasets. Compared to the alignment-based methods, this alternative method has complementary advantages, for example, to identify the novel protein sequences with remote homology to their known counterparts. Additionally, our method could also be applied for identifying the other protein sequences with the characteristic of high complexity and low conservation, where it would be another interesting way to explore.

##  Supplemental Information

10.7717/peerj.13404/supp-1 Supplemental Information 1 The length distribution of the three protein sequences

10.7717/peerj.13404/supp-2 Supplemental Information 2 The Venn diagrams of the prediction results of three methods (*i.e.,* DeephageTP, Diamond, and HMMER) on the metagenomic datasets ERR2868024: A(TerL), B(Portal), C(TerS); SRR7892426: D(TerL), E(Portal), F(TerS).

10.7717/peerj.13404/supp-3 Supplemental Information 3 Supplemental Tables

## Additional Information and Declarations

### Data Availability

The following information was supplied regarding data availability: The code and data are available at GitHub: https://github.com/chuym726/DeephageTP.
