# Supplementary figures and images for: DeephageTP: a convolutional neural network framework for identifying phage-specific proteins from metagenomic sequencing data
Source: PeerJ. 2022 Jun 8;10:e13404. doi: 10.7717/peerj.13404 (PMC9188312; doi:10.7717/peerj.13404)

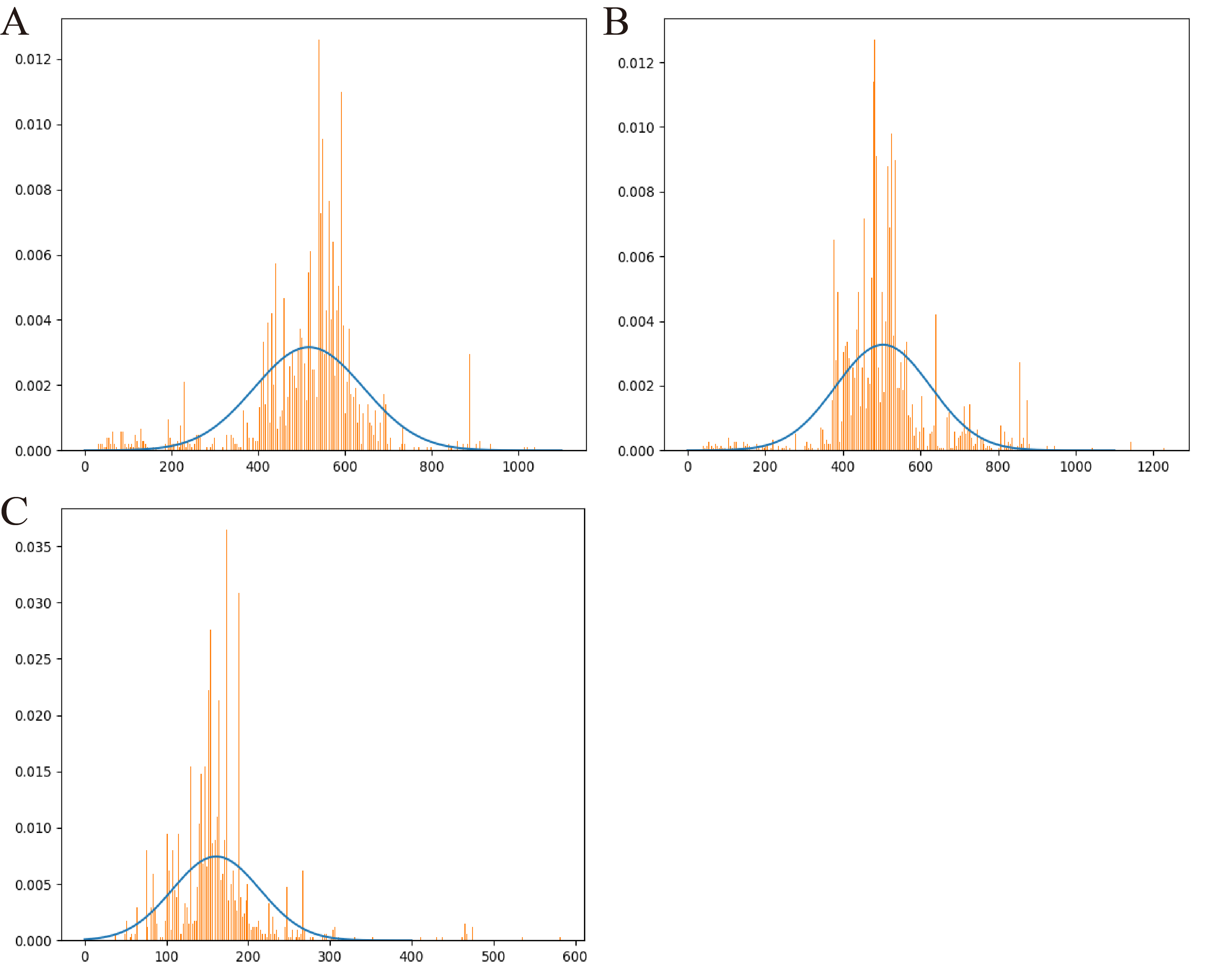

Supplement: Supplemental Information 1 [file peerj-10-13404-s001.png]

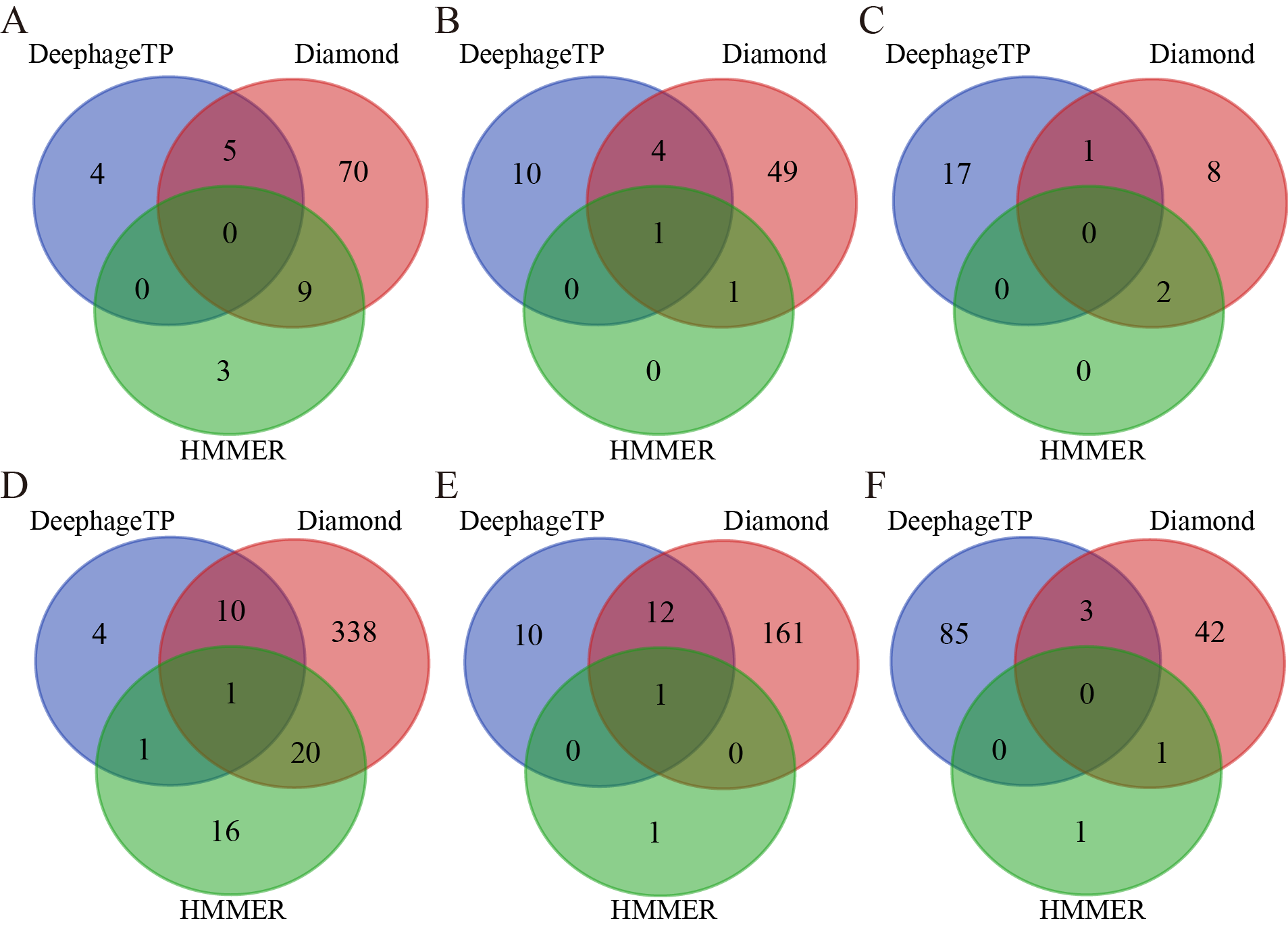

Supplement: Supplemental Information 2 — ERR2868024: A(TerL), B(Portal), C(TerS); SRR7892426: D(TerL), E(Portal), F(TerS). [file peerj-10-13404-s002.png]
